# A globally-distributed alien invasive species poses risks to United States imperiled species

**DOI:** 10.1038/s41598-018-23657-z

**Published:** 2018-03-28

**Authors:** Meredith L. McClure, Christopher L. Burdett, Matthew L. Farnsworth, Steven J. Sweeney, Ryan S. Miller

**Affiliations:** 1 Conservation Science Partners, Truckee, California, United States; 2 Department of Biology, Colorado State University, Fort Collins, Colorado, United States; 3 Center for Epidemiology and Animal Health, Animal and Plant Health Inspection Service, United States Department of Agriculture, Fort Collins, Colorado, United States

## Abstract

In the midst of Earth’s sixth mass extinction event, non-native species are a driving factor in many imperiled species’ declines. One of the most widespread and destructive alien invasive species in the world, wild pigs (*Sus scrofa*) threaten native species through predation, habitat destruction, competition, and disease transmission. We show that wild pigs co-occur with up to 87.2% of imperiled species in the contiguous U.S. identified as susceptible to their direct impacts, and we project increases in both the number of species at risk and the geographic extent of risks by 2025. Wild pigs may therefore present a severe threat to U.S. imperiled species, with serious implications for management of at-risk species throughout wild pigs’ global distribution. We offer guidance for efficient allocation of research effort and conservation resources across species and regions using a simple approach that can be applied to wild pigs and other alien invasive species globally.

## Introduction

The Earth is experiencing a sixth mass extinction event, with current rates of species loss far exceeding the background extinction rate^1^. More than 25% of known species remaining in the wild are classified as critically endangered, endangered, or vulnerable^2^. These declines are largely driven by human activities, including destruction or alteration of habitat, overharvest, and the spread of harmful non-native species^3^.

A strategic plan adopted by parties to the Convention on Biological Diversity (CBD) set global targets for preventing extinction of known imperiled species (Target 12) and expanding protection and effective management of sites with biodiversity conservation significance (Target 11)^4^. The total annual cost of meeting these targets is estimated at U.S. $76.1 billion^5^, of which only 12% is currently funded. In the United States (U.S.), the Endangered Species Act (ESA) of 1973 aims to protect threatened and endangered (hereafter ‘imperiled’) species, along with the ecosystems on which they depend. The ESA provides for designation of critical habitat, species management programs, and penalties for knowingly harming or trafficking imperiled species. In 2014, the U.S. Fish and Wildlife Service (USFWS) and other agencies spent over U.S. $1.4 billion on recovery efforts for 1,600 currently listed species^6^.

Alien invasive species constitute a major environmental stressor^7,8^ and are considered to be a driving factor in the decline of 3,862 species (16.6%) globally listed as extinct or imperiled^2^. In the U.S., non-native species are considered the primary cause of decline in 42% of imperiled species via habitat alteration, competitive displacement, predation, spread of pathogens, and hybridization^9^.

Wild pigs (*Sus scrofa*), also known as feral hogs, feral swine, or wild boar, are recognized as one of the most widespread and destructive invasive species in the world^10–12^. Wild pigs are native to Eurasia and Northern Africa, but have been widely introduced for centuries, often deliberately, and now occupy every continent except Antarctica^13,14^. They are extreme generalists in habitat and diet^10^, reproduce prolifically^15^, transmit a wide array of parasites and diseases^16–19^ and are considered ecosystem engineers^20–22^ due to their habit of rooting soils, resulting in high capacity to negatively impact a wide variety of native species (Table 1).

**Table 1:** Criteria for selecting threatened and endangered species considered susceptible to direct impacts of wild pigs where they co-occur.

Taxon	Susceptible subset	Impact mechanism	References
Amphibians	All	Predation on individuals, destruction of habitat	^35, 44– 49^
Birds	Ground-nesting	Predation on eggs and/or individuals, destruction of habitat	^35, 46– 48, 50– 64^
Crustaceans	Shallow, slow-water, and/or mud substrate freshwater habitat	Predation on individuals, potential destruction of habitat	^50, 55, 57, 65, 66^
Mammals	Ground-dwelling, burrowing, tunneling	Predation on individuals, destruction of habitat	^46– 48, 56, 62, 67^
Mollusks	Shallow, slow-water, and/or mud substrate freshwater habitat	Predation on individuals, potential destruction of habitat	^15, 57, 65, 66, 68^
Reptiles	All	Predation on eggs and/or individuals, destruction of habitat	^35, 46– 49, 56, 62, 66, 69– 73^

**Figure 1:**
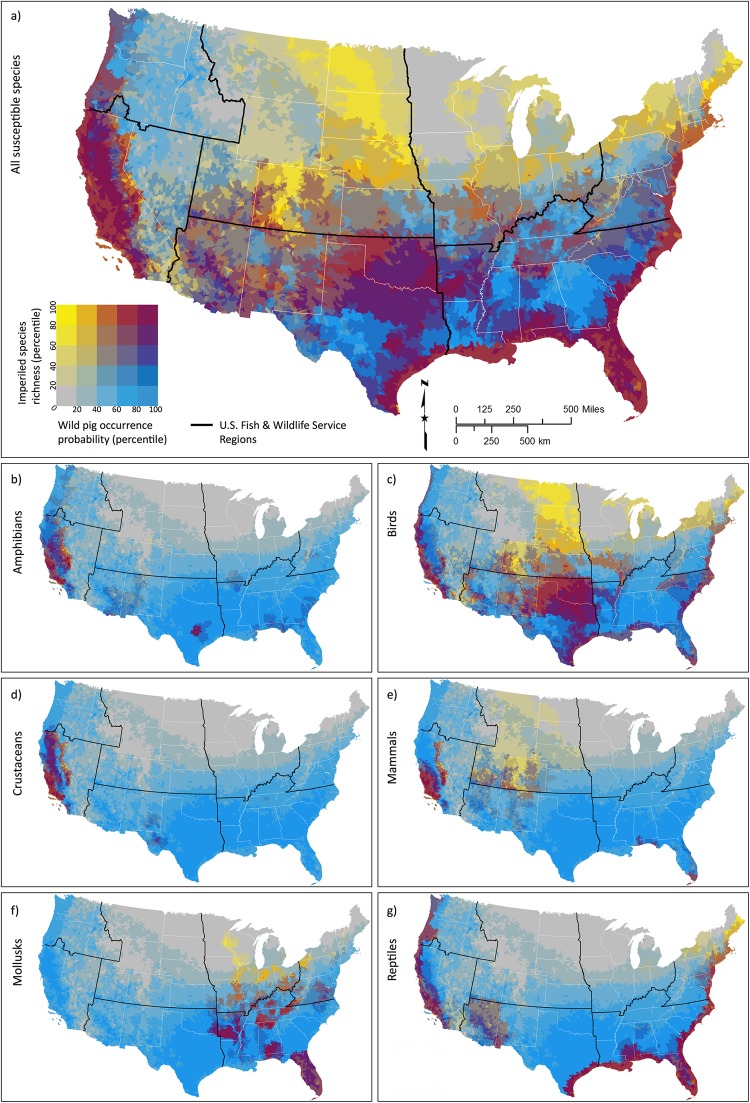
Regions of the contiguous U.S. currently occupied by wild pigs have among the highest numbers of threatened and endangered species expected to be susceptible to impacts from wild pigs (Table 1). Susceptible threatened and endangered species richness versus wild pig probability of occurrence^25^ across the contiguous U.S. for (**a**) all susceptible species and by taxa: (**b**) amphibians, (**c**) birds, (**d**) crustaceans, (**e**) mammals, (**f**) mollusks, and (**g**) reptiles^42^.

Wild pigs were introduced to what are now the southeast United States and California by Spanish explorers and settlers in the 16^th^ and 18^th^ centuries, respectively^23^. They are now the most abundant free-ranging, non-native ungulate in North America^24^. Recent work predicting the relative probability of wild pig occurrence across the contiguous U.S. based on their physiology and ecology^25^ suggests that many areas of the country not yet colonized by wild pigs have a high probability of meeting habitat requirements and supporting future population establishment of wild pigs. Furthermore, regions currently occupied by wild pigs and those with the highest invasion potential^25,26^ are among the most biodiverse in the contiguous U.S. (Fig. 1). The U.S. Department of Agriculture (USDA) invests $20 million annually to manage wild pig damage to agricultural and natural resources, property, animal health, and human health and safety^27^.

Annual costs of controlling invasive species occupying habitats of U.S. endangered species have been estimated to total $29–38 million (1997 $U.S.), and without this continued level of support, 60% of these species could be subject to declines or extinction, even if their habitats are nominally protected^28^. Given the high costs of managing imperiled and invasive species, along with the potential for alien invasives to necessitate and/or undermine ongoing imperiled species recovery efforts^9,29^, it is imperative that managers understand the scope of invasives’ potential impacts on at-risk species. Even coarse-scale impact estimates are expected to help managers to identify taxa and regions that may be most at risk and allocate research and management resources effectively. Yet despite their global introduced distribution and highly destructive nature, invasive wild pigs are remarkably understudied and poorly understood in terms of their impacts on native imperiled species.

We present estimates of current and future risks of direct wild pig impacts on imperiled species across the contiguous U.S. based on their current and projected species range overlap^30,31^. To our knowledge, the potential impacts of this incredibly widespread and destructive invasive species on at-risk native species have not yet been estimated anywhere within their introduced range. Our first approximation of the potential scope of wild pig threats to imperiled species - given the current state of knowledge and available data - offers an important starting point for effective allocation of efforts to reduce extinction risks to taxa and regions most heavily impacted by wild pigs in the context of other threats (e.g., habitat conversion, climaate change, other invasives). This assessment can inform targeted management of wild pig spread, as well as efficient allocation of efforts toward more detailed assessment of impact risks. Our simple approach can easily be extended throughout wild pigs’ global range and is readily applicable to estimating potential impacts of many other invasive species.

## Results

### Current risk

**Figure 2:**
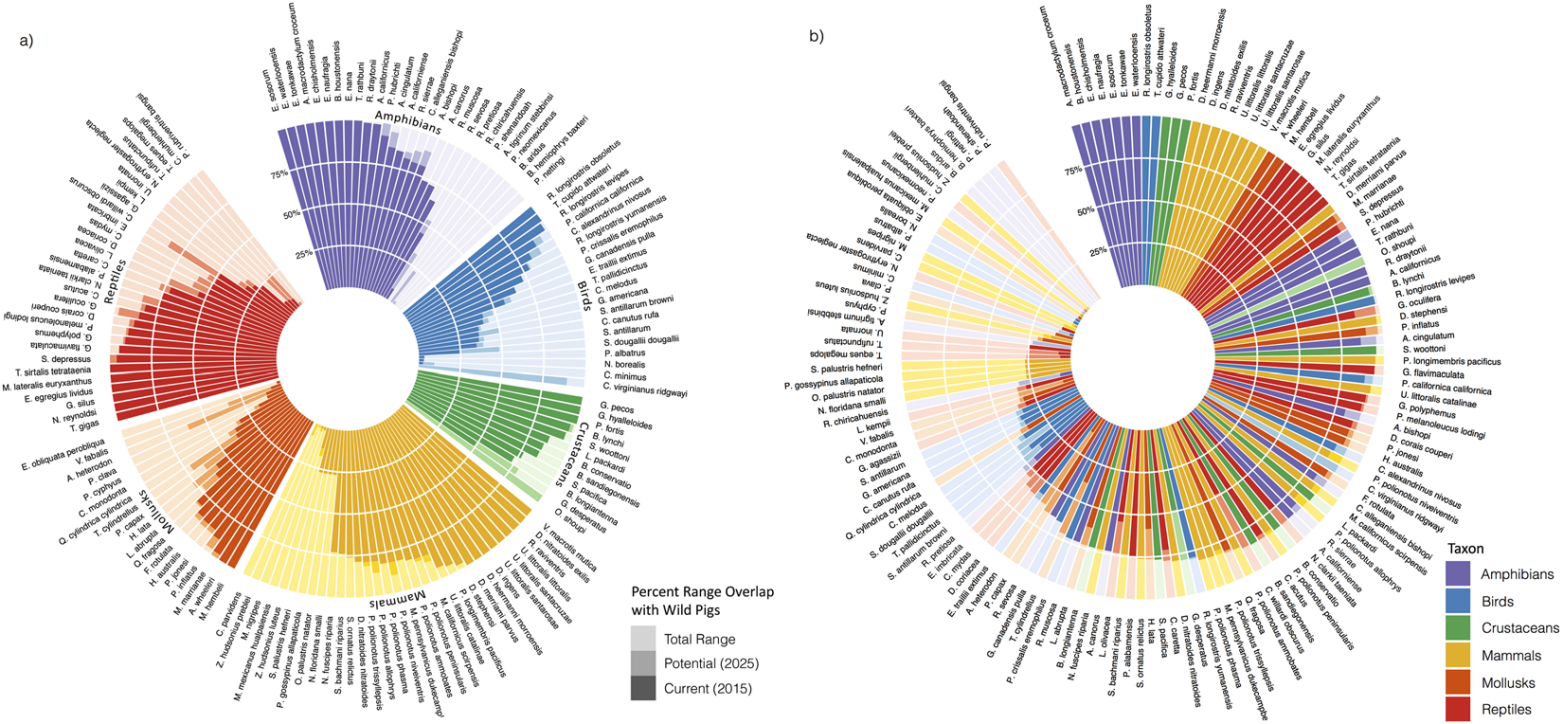
Most threatened and endangered species in the contiguous U.S. expected to be susceptible to wild pig impacts have extensive range overlap with wild pigs that is projected to increase by 2025. Proportion of each susceptible species’ range currently occupied by wild pigs and estimated to potentially be occupied by 2025, displayed (**a**) by taxonomic group and (**b**) by decreasing proportion of the range that is currently occupied^43^.

There are a total of 284 imperiled species in the animal taxa we considered (see Methods), of which we identified 141 species expected to be susceptible to wild pig impacts if exposed (Table 1, Supplementary Dataset). Of these species, we estimate that 123 (87.2%) may currently be at risk, with mean wild pig range overlap of 72.7% (29.0% SD) (Fig. 2, Table 2). Crustaceans may be most at risk: eleven of 12 crustacean species co-occur with wild pigs, with a mean of 86.3% (15.5% SD) range overlap. Nearly all mollusks (18 of 19) also co-occur with wild pigs, but mean range overlap is relatively low (58.3 ± 33.9% SD).

**Table 2:** Summary of current (2015) and estimated potential (2025) wild pig range overlap with susceptible threatened and endangered species in the contiguous U.S., across taxa and regions.

	Current Impacts (2015)		Potential Impacts (2025)	
	*Species* (*n/total*)	*Percent Range* (*μ* ± *SD*)	*Additional Species* (*CI*)	*Percent Range Increase* (*μ* ± *SD*)
All	123/141	72.7% + /−29.0%	5 (1–11)	6.1% + /− 14.0%
Endangered	87/95	72.7% + /−28.3%	3 (1–8)	6.3% + /−16.3%
Threatened	36/46	72.5% + /−30.8%	2 (0–3)	5.8% + /−7.7%
Amphibians	22/28	85.0% + /−22.8%	1 (0–4)	4.1% + /−7.2%
Birds	16/20	62.5% + /−26.5%	2 (1–2)	9.2% + /−20.8%
Crustaceans	11/12	86.3% + /−15.5%	1 (0–1)	11.0% + /−29.3%
Mammals	29/33	78.0% + /−26.8%	1 (0–3)	1.2% + /−2.4%
Mollusks	18/19	58.3% + /−33.9%	0 (0–0)	10.3% + /−14.0%
Reptiles	27/29	66.9% + /−32.2%	0 (0–1)	6.3% + /−8.6%
Pacific region	10/12	44.3% + /−39.5%	1 (0–1)	22.4% + /−21.6%
Southwest region	36/44	83.0% + /−30.1%	4 (1–8)	9.7% + /−17.4%
Midwest region	8/17	21.9% + /−24.1%	1 (0–3)	1.0% + /−2.1%
Southeast region	57/58	84.7% + /−18.8%	1 (0–1)	10.3% + /−17.9%
Northeast region	8/20	26.8% + /−19.6%	1 (0–8)	11.9% + /−18.0%
Mountain Prairie region	3/21	41.5% + /−25.5%	4 (1–10)	11.7% + /−21.4%
Pacific Southwest region	52/55	86.5% + /−19.6%	0 (0–1)	0.4% + /−1.1%

We estimate that risks are greatest in USFWS Southeast and Pacific Southwest regions (Fig. 1, Table 2). Wild pigs are most pervasive in the Southeast (72.9% of region), co-occurring with 57 imperiled species with an average range overlap of 84.7% (18.8% SD). Wild pigs occupy 41.3% of the Pacific Southwest, co-occurring with 52 imperiled species with an average range overlap of 86.5% (19.6% SD). The Southwest is also highly at risk, with 62.6% wild pig occupancy overlapping the ranges of 36 imperiled species by an average of 83.0% (30.1% SD).

### Future risk

**Figure 3:**
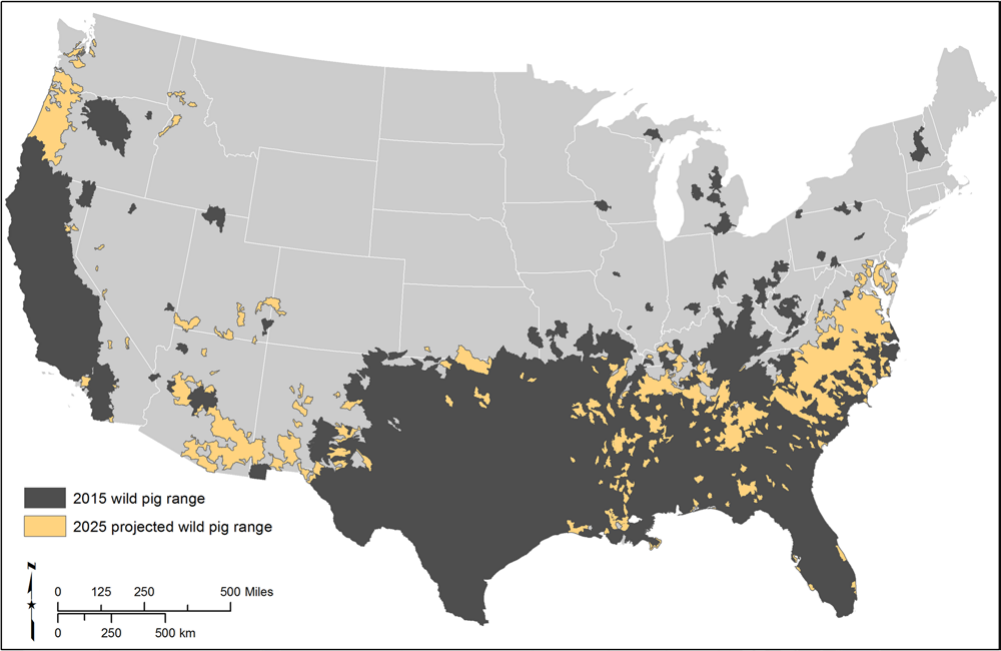
Wild pigs are projected to continue expanding their contiguous U.S. range through 2025. Current (dark gray) and projected 2025 (orange) geographic range of wild pigs in the contiguous U.S^42^.

If wild pigs continue their current rate of spread across the contiguous U.S., more imperiled species are likely to be at risk across a greater portion of their range. We estimate that 1,036 (CI: 273–1,800) additional watersheds may be occupied by wild pigs by 2025, an annual increase of 2.17% in the number of occupied watersheds (Fig. 3). In this scenario, 128 (90.8%) (CI: 87.9–95%) of 141 susceptible imperiled species may co-occur with wild pigs in at least 5% of their range, with a mean of 76% (27.1% SD) range overlap (Fig. 2, Table 2). This represents 5 (CI: 1–11) additional imperiled species potentially at risk, and a 6.1% (14.0% SD) mean increase in the extent of species’ pig-occupied ranges.

We expect crustaceans to continue to be the most at-risk taxon, with all (12) susceptible species potentially co-occurring with wild pigs by 2025 and a mean increase in range overlap of 11.0%, resulting in an overall mean range overlap of 90.1% (10.1% SD). Birds are expected to experience the greatest increase (10%) in number of species co-occurring with pigs (2 additional species), accompanied by a 9.2% mean increase in range overlap. Although we don’t predict any additional mollusk species to be exposed to pigs by 2025, we expect those currently at risk to experience a mean 10.3% increase in range overlap. Mammals are expected to have the smallest increase in future risk, with one additional species potentially exposed and a mean 1.2% increase in range overlap.

We predict wild pigs’ range to expand most in the already at-risk Southeast, where watershed occupancy could increase by 18.3% (CI: 6.8–23.8%) to reach a total occupancy level of 91.2% by 2025 (Fig. 1, Table 2). Mean range overlap is expected to increase by 10.3% and one additional species may be placed at risk, with most imperiled species in the Southeast already co-occurring with wild pigs. We also predict substantial range expansion in the Northeast, Southwest, and Pacific, with 8.6%, 7.4%, and 6.4% increases in wild pig occupancy, respectively. Of these regions, expansion in the Southwest may place the most additional species at risk (4 species; CI: 1–8 species), while expansion in the Pacific region may result in the greatest mean increase in wild pig range overlap with imperiled species (22.4%).

## Discussion

Wild pigs are highly invasive and destructive generalists that occupy much of the contiguous U.S. and have high potential for further spread^24,25^. We show that wild pigs already co-occur with most imperiled species in the contiguous U.S. that are susceptible to direct impacts, and that wild pigs tend to occupy the majority of those species’ ranges. We predict the risks of wild pig impacts to increase if pigs continue to expand their range as projected over the next 10 years, both in terms of the number of imperiled species at risk and the extent of these species’ ranges in which risks occur.

Wild pigs inflict a variety of documented impacts on native animal species (Table 1), with likely impacts on other species occupying similar niches. Their large body size and omnivorous, highly plastic diet provides access to a wide range of animal prey, including all terrestrial vertebrate classes, eggs of reptiles and ground-nesting birds, soil invertebrates, and shallow freshwater invertebrates, with animal matter accounting for up to 30% of wild pigs’ diet^10,32^ (Table 1). Their rooting and wallowing behavior results in marked ecosystem-level impacts through alteration of soil properties as well as plant community structure and composition^10,22^, destroying habitat for a wide variety of species, particularly tunneling, burrowing, and litter-dwelling species (Table 1). Our assessment suggests that impacts of wild pigs via these mechanisms on U.S. imperiled species could be extensive.

The true magnitude of wild pigs’ impacts may be much greater when other, less-studied impact mechanisms are considered. Pigs are known carriers of diverse parasites and other disease-causing pathogens, with potential to transmit pathogens to both livestock and wildlife^18,19^. Prevalence varies geographically and among pathogens^24^, and transmission dynamics are not well understood, but impacts on imperiled species can be serious^33^. Wild pigs may also compete with native species for resources^10^. Disease transmission and resource competition may not only exacerbate other impacts on imperiled species, but also increase the total number of species susceptible to wild pig impacts. Potential impacts on medium- to large-bodied mammals may be particularly underestimated due to exclusion of these mechanisms^10^.

In the absence of detailed information about imperiled species’ distributions within their ranges or species-specific interactions with wild pigs, we assumed that species with attributes similar to those for which impacts have been documented may be at risk wherever they co-occur with wild pigs^30,31^. Due to the coarse scale of the datasets available for our analysis, we recognize that imperiled species and wild pigs may not co-occur at finer spatial scales within all watersheds suggested to be co-inhabited. Certainly, some imperiled species may use different habitats than wild pigs within the watersheds where they do co-occur and therefore may not interact directly with pigs. However, because data to assess co-occurrence at finer scales are unavailable, we suggest that our preliminary assessment based on existing data provides a much-needed first approximation of the potential severity of current and future risks of wild pig impacts on imperiled species in the U.S. Despite some likely error in predictions at the individual species level, the patterns we elucidate in relative potential risk among taxa and regions provide critical initial guidance for effective allocation of management efforts as well as efficient investment in finer-scale assessment of wild pig co-occurrence with and impacts on at-risk species. Furthermore, we suggest that impacts in regions of even broad overlap are likely given pigs’ extreme habitat generalism, diverse diet, and highly destructive rooting habits. Wild pigs can also shift foraging strategies in response to temporal variation in food resources and to opportunistically target highly concentrated prey, and their rooting behavior has been suggested to disproportionately impact specialist species^34^. This can endanger species that are dependent on highly specialized habitat types or ephemeral conditions despite their overall rarity across the landscape^35^.

We also believe that our projection of wild pigs’ potential distribution by 2025 is likely a conservative one. We project occupancy of 35% of watersheds in the contiguous U.S. by 2025, yet eventual spread of wild pigs to all watersheds where probability of occurrence^25^ is equal to or greater than that of watersheds where they already occur would result in 93.7% occupancy. Other recent work similarly predicts wild pig expansion throughout most of the contiguous U.S. in the next 3–5 decades^26^. Furthermore, limitations on pig establishment and density imposed by cold temperatures at northern latitudes and higher elevations^25^ may be relaxed as climate change results in fewer days with extreme cold temperatures and less snow in these regions, allowing more extensive occupancy and/or occupancy at higher densities^26,36^. In addition, we have only considered likely patterns of spread by natural dispersal mechanisms, but wild pigs have been introduced to new regions of the U.S. far from their historic range for hunting or other purposes; we expect this practice to continue.

Expenditures on management of the 123 imperiled species that currently co-occur with wild pigs totaled $182.2 million in 2014^6^. If wild pigs continue to spread as projected, their impacts could become an additional factor in the management of up to 11 additional species for which 2014 expenditures totaled $8.56 million. An important goal for the USDA’s wild pig management program is to predict wild pig population expansion and associated risk to animal health^37^, so that wild pig and imperiled species management activities can be more effectively prioritized and coordinated. The program typically focuses on wild pig damage reduction where populations are well-established, but also partners with state and federal agencies to eliminate wild pigs where their populations are low or where they are just beginning to establish. We show where estimation and reduction of damage from established pigs may benefit the greatest number of imperiled species and where emerging populations may be most critical to control. Prioritizing efforts based on these findings may help minimize future expenditures on both wild pig and imperiled species management. Prioritization of these strategies should also be considered in the context of additional threats to imperiled species, such as habitat conversion, impacts of other invasive species, and climate change.

Our simple and proactive approach to estimating potential risks is modeled after similar invasive species assessments conducted in other systems in which limited information is available^30,31^, and it can easily be extended to other portions of wild pigs’ global introduced range or used to assess risks from other invasive species. This approach is repeatable and comparable across taxa, and provides a starting point for understanding the relative impacts of invasive species on imperiled species in the context of other stressors (e.g., additional non-native species, overexploitation, habitat loss and degradation, and climate change^31^). Similar assessments of the spatial distribution of these stressors, combined with information about mechanisms by which they impact imperiled species, could reveal where imperiled species may be most vulnerable to the cumulative effects of multiple stressors and inform coordinated management actions to comprehensively address these threats. Understanding the scope and distribution of alien invasive species’ potential impacts on native imperiled species, given available data, is a crucial first step to managing risks, identifying research priorities, and allocating conservation resources effectively.

## Methods

We selected imperiled species expected to be susceptible to wild pig impacts based on published observations of wild pig interactions with other taxa (Table 1). We focused our assessment on animal species (excluding most arthropods) because little is known about the mechanisms by which wild pigs impact plant, fungus, insect, and arachnid species. We focused on risks related to directly observable impacts (i.e., predation, habitat destruction) because the prevalence and severity of other mechanisms (i.e., competition, disease spread) are not well understood. Our assessment thus conservatively estimates the total number of species at risk.

We obtained geographic range data for animal species designated as threatened or endangered in the U.S. from USFWS. Because USFWS manages imperiled species, these range estimates were the most authoritative source available for the U.S. and were more precise than estimates from global sources (e.g., IUCN). However, quality and resolution varied among species and, in some cases, among portions of a species’ range spanning multiple USFWS regions. Most species’ ranges (83.7%) were delineated in whole or in part at the county level. Many (42.2%) were delineated in whole or in part as polygons drawn by USFWS field office biologists. Other less frequently used units of delineation include HUC8^38^ watersheds and USGS 24 K quadrangle or quarter-quadrangle grids^39^.

We estimated the current distribution of wild pigs in the contiguous U.S. using data from the National Feral Swine Mapping System^40^, aggregated to watersheds (HUC10^38^) as described in McClure *et al*.^25^. This dataset has been compiled at irregular intervals since 1960 and annually since 2008. Map polygons representing the known geographic extent of established wild pig populations (i.e., present for two or more years and evidence of reproduction) were reported nationally by wildlife professionals in state wildlife resource agencies and the United States Department of Agriculture. Polygon locations were drawn manually using topographical maps to reference areas where pigs have been observed to meet criteria for establishment. These polygons were aggregated to watersheds (HUC10) as described in McClure *et al*.^25^ to discretize consistent, comparable, ecologically relevant sampling units.

We estimated wild pigs’ potential future distribution using observed trends in past spread and a previously published model of wild pig occurrence probability^25^ as follows. We estimated the proportion of wild pig-occupied watersheds at intervals for which data were available from 1960 to 2015, then modeled the trajectory of change in the proportion of occupied watersheds over time. Using an information theoretic approach^41^, we selected a 3-parameter logistic regression model, which we used to predict the proportion of occupied watersheds in the year 2025 (Supplementary Table S1, Figure S1). Based on this proportion, and assuming that all currently occupied watersheds remain occupied in 2025, we determined the number of additional currently unoccupied watersheds predicted to be occupied by 2025. We then populated this number of unoccupied watersheds to estimate future wild pig distribution.

The spatial distribution of these additional watersheds was selected based on the assumption that watersheds most likely to be occupied in the future were those modeled to have the greatest probability of wild pig occurrence by McClure *et al*.^25^ (Fig. 3). This existing model used a logistic discrimination function to estimate the relative probability of wild pig occurrence across the contiguous U.S. given the distribution of habitat covariates at watersheds in which pigs are present relative to randomly sampled ‘background’ watersheds. This approach was selected because it avoids the problematic assumption that background watersheds represent absences or ‘pseudo-absences’ of wild pigs. The inferential model, which was selected within an information-theoretic framework using multi-model inference^41^, includes environmental covariates customized to represent known physiological and ecological constraints on wild pig distributions. These include mean number of days with temperatures above 35 °C; mean number of days with temperatures below −4 °C; mean snow depth on April 1; mean distance to nearest perennial stream or water body perimeter; percent area with forest cover; forage availability, quantified as percent area with mast-producing or crop cover; and habitat heterogeneity, quantified as the mean number of key habitat elements (water, cover, and forage) available within a radius defined by an average home range size. Cross-validation of model results indicated strong predictive capacity (Pearson’s *r* = 0.989). Watersheds with the highest estimated probability of occurrence tended to be immediately adjacent to the periphery of wild pigs’ current distribution, and were therefore considered to represent a reasonable prediction of the pattern of future spread by natural dispersal mechanisms; we do not consider the potential for human introductions of wild pigs to novel areas for hunting or other purposes here.

In a geographic information system (GIS), we intersected imperiled species’ ranges with the current and potential range of wild pigs to estimate the number of species currently or potentially at risk of wild pig impacts and the proportion of each species’ range at risk^30,31^. Only species with at least 5% of their ranges occupied by wild pigs were considered to be potentially at risk, although our results were not sensitive to this threshold as most species had far more extensive range overlap (see Results). Confidence limits on estimates were derived from the confidence interval on the proportion of watersheds projected to be occupied in 2025. Each estimate was further broken down by taxonomic group and USFWS region.

### Data availability

The original wild pig occurrence data and threatened and endangered species range data used in this analysis cannot be made available in the manuscript because they were obtained from third party providers. The data providers are not able to share the spatial data publicly due to sensitivities around the location of these species. Threatened and endangered species may be subject to exploitation, and there is a history of hunters using the wild pig data to locate populations then bait, trap, and relocate them for hunting purposes, which greatly interfered with ongoing control and eradication efforts. Threatened and endangered species range data are available by request from the U.S. Fish and Wildlife Service (Mark Saunders, mark_saunders@fws.gov). Wild pig occurrence data are available by request through the Southeastern Cooperative Wildlife Disease Study (SCWDS) National Feral Swine Mapping System (http://swine.vet.uga.edu/nfsms/; Joe Corn, feralpig@uga.edu).

## Electronic supplementary material

Supplementary Material

Dataset 1

High Definition Figure 2
