# Publisher Correction: A globally-distributed alien invasive species poses risks to United States imperiled species

**DOI:** 10.1038/s41598-018-26014-2

**Published:** 2018-07-19

**Authors:** Meredith L. McClure, Christopher L. Burdett, Matthew L. Farnsworth, Steven J. Sweeney, Ryan S. Miller

**Affiliations:** 1 Conservation Science Partners, Truckee, California, United States; 2 Department of Biology, Colorado State University, Fort Collins, Colorado, United States; 3 Center for Epidemiology and Animal Health, Animal and Plant Health Inspection Service, United States Department of Agriculture, Fort Collins, Colorado, United States

Correction to: *Scientific Reports* 10.1038/s41598-018-23657-z, published online 28 March 2018

In the original version of this Article, Matthew L. Farnsworth was incorrectly affiliated with “Department of Biology, Colorado State University, Fort Collins, Colorado, United States”. The correct affiliation is listed below:

“Conservation Science Partners, Truckee, California, United States”

This error has now been corrected in the PDF and HTML versions of the Article. In addition, the HTML version of this Article contains a lower resolution Figure 2.

The fully detailed version of this figure is now available to download as a Supplementary Information File.

