# Supplementary material for: A globally-distributed alien invasive species poses risks to United States imperiled species
Source: Sci Rep. 2018 Mar 28;8:5331. doi: 10.1038/s41598-018-23657-z (PMC5871849; doi:10.1038/s41598-018-23657-z)
Supplement: Supplementary file 1 — Supplementary Material [file 41598_2018_23657_MOESM1_ESM.pdf]

A globally-distributed alien invasive species poses risks to United States imperiled species

Meredith L. McClure*, Conservation Science Partners, Truckee, California, United States

Christopher L. Burdett, Department of Biology, Colorado State University, Fort Collins, Colorado, United States

Matthew L. Farnsworth, Conservation Science Partners, Truckee, California, United States

Steven J. Sweeney, Center for Epidemiology and Animal Health, Animal and Plant Health Inspection Service, United States Department of Agriculture, Fort Collins, Colorado, United States

Ryan S. Miller*, Center for Epidemiology and Animal Health, Animal and Plant Health Inspection Service, United States Department of Agriculture, Fort Collins, Colorado, United States

Past and Projected Spread of Wild Pigs by Watershed

Table S1. Model selection results for the trajectory of change in proportion of watersheds occupied by wild pigs in the contiguous U.S. from 1960 to 2015.

Model	*df*	*AIC*	*dAIC*	*AIC weight*
Linear	3	-50.616	11.156	3.53E-03
Exponential	3	-10.930	50.842	8.52E-12
Power	3	-10.690	51.082	7.56E-12
Gamma	3	-56.343	5.429	0.062
Logistic (3-parameter)	3	-61.772	0	0.935

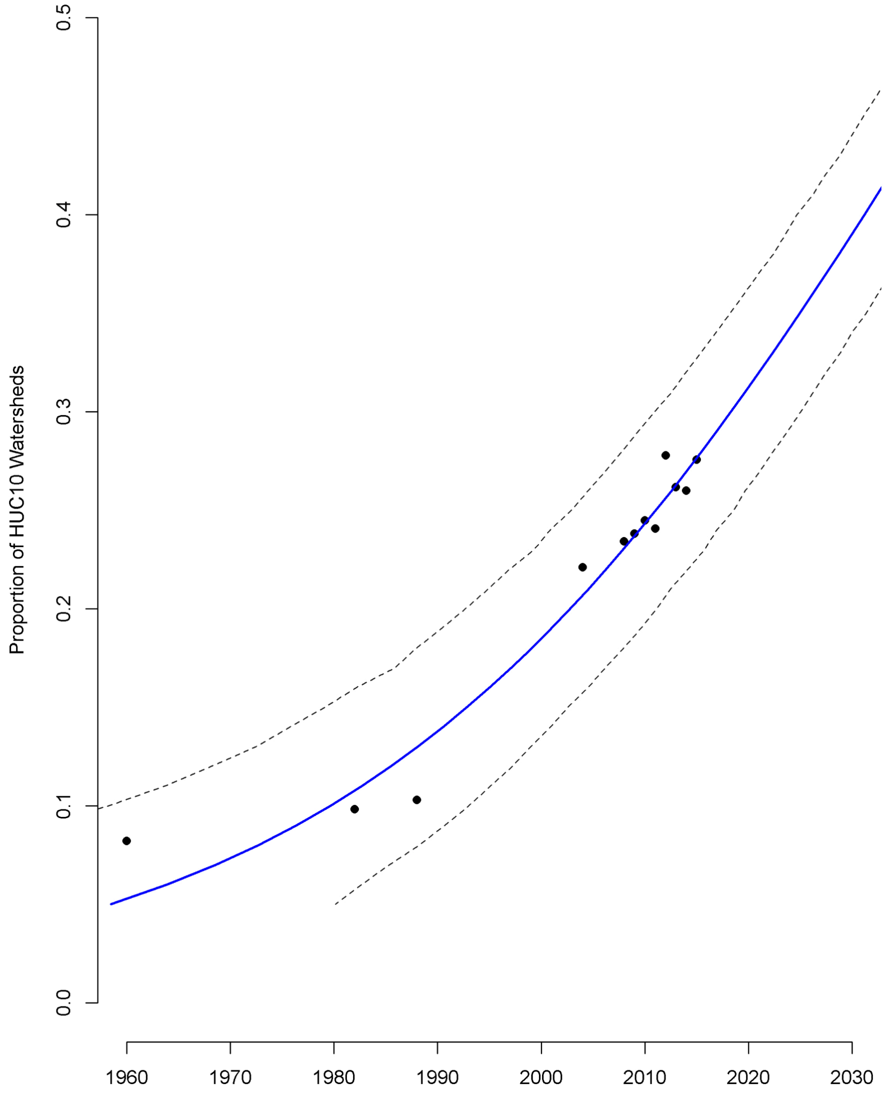

Figure S1. Change in proportion of watersheds in the contiguous U.S. occupied by wild pigs from 1960 to 2015, with projected occupancy from 2015 to 2030. A 3-parameter logistic regression model was fitted (blue line; table below) along with a 95% confidence interval (dotted lines).
