# Supplementary figures and images for: A globally-distributed alien invasive species poses risks to United States imperiled species
Source: Sci Rep. 2018 Mar 28;8:5331. doi: 10.1038/s41598-018-23657-z (PMC5871849; doi:10.1038/s41598-018-23657-z)

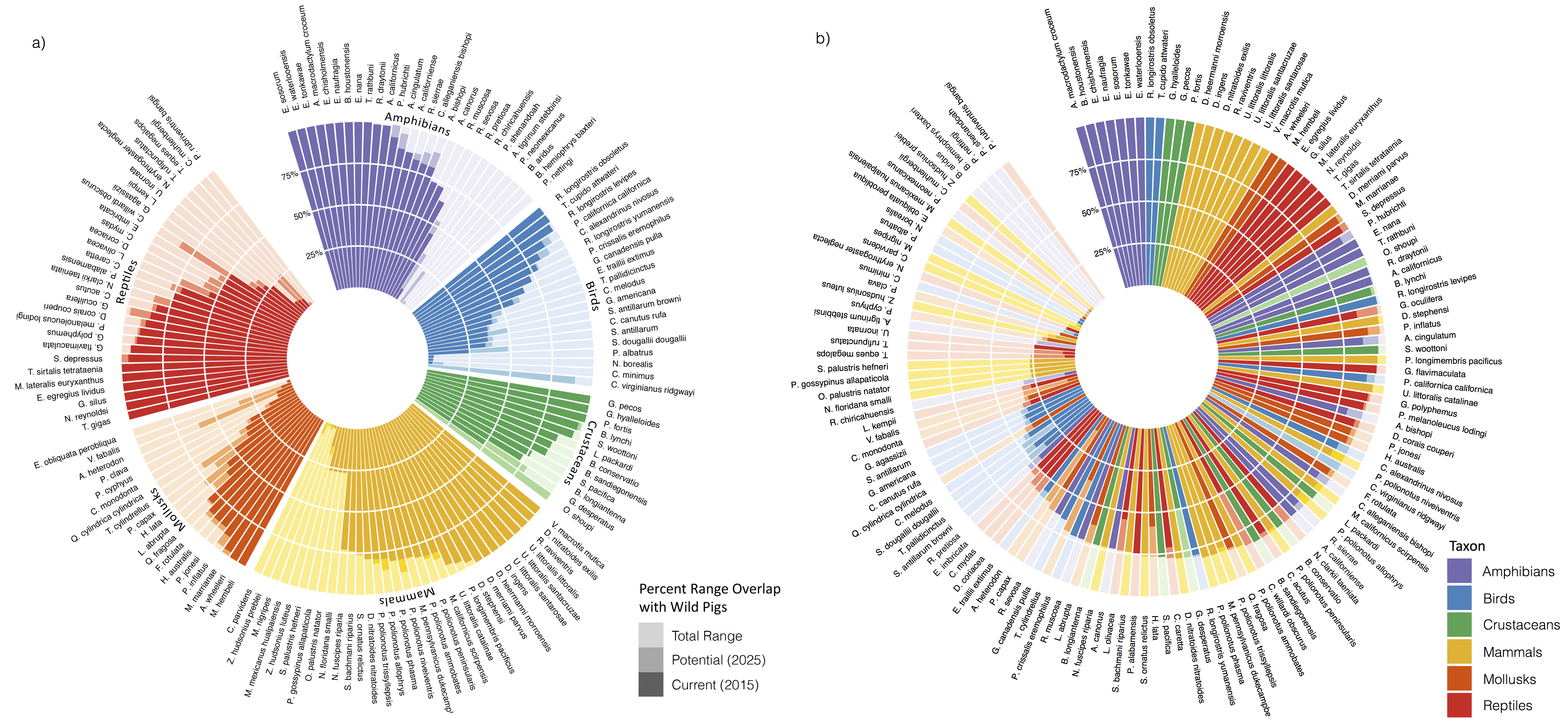

Supplement: Supplementary file 3 — High Definition Figure 2 [file 41598_2018_23657_MOESM3_ESM.tif]
